# Validation of International Working Group response criteria in higher‐risk myelodysplastic syndromes: A report on behalf of the MDS Clinical Research Consortium

**DOI:** 10.1002/cam4.3608

**Published:** 2020-12-22

**Authors:** Rami S. Komrokji, Najla H Al Ali, David Sallman, Eric Padron, Amy E. DeZern, John Barnard, Gail J. Roboz, Guillermo Garcia‐Manero, Alan List, David P. Steensma, Mikkael A. Sekeres

**Affiliations:** ^1^ Malignant Hematology Department H Lee Moffitt Cancer Center and Research Institute Tampa FL USA; ^2^ Kimmel Cancer Center/Johns Hopkins University Baltimore MD USA; ^3^ Department of Quantitative Health Sciences Cleveland Clinic Cleveland OH USA; ^4^ Weill Cornell Medical College New York NY USA; ^5^ Department of Leukemia MD Anderson Cancer Center Houston TX USA; ^6^ Dana‐Farber Cancer Institute Boston MA USA; ^7^ Leukemia Program Cleveland Clinic Cleveland OH USA

**Keywords:** high‐risk disease, international working group, myelodysplastic syndromes, response criteria

## Abstract

The utility of the International Working Group (IWG) 2006 response criteria for myelodysplastic syndromes (MDS) as a surrogate endpoint for outcomes is unclear. We assessed the validity of the IWG 2006 response criteria in a large cohort of higher‐risk MDS patients (pts) treated at centers from the MDS Clinical Research Consortium. The best overall response rate (ORR) by IWG 2006 criteria to first‐line therapy among 597 evaluable pts was 38% and include complete response (CR) 16%, marrow CR (mCR) 2%, partial response (PR) 10%, hematological improvement (HI) 10%, stable disease (SD) 33%, and progressive disease (PD) 24%. CR was associated with a better overall survival (OS) compared to all other response groups (*P* < 0.001). Among 470 pts treated with hypomethylating agent (HMA) as first‐line therapy, the overall Response Rate, defined as HI or better was 39%. The median OS from time of best response was 21 mo, 8 mo, 14 mo, 12 mo, 13 mo, and 8 mo for CR, mCR, PR, HI, SD, and PD, respectively (*P* < 0.001). We validated those results in a separate cohort of 539 higher‐risk MDS pts treated at Moffitt Cancer Center who received first‐line HMA therapy, particularly addressing the value of mCR and mCR+HI. mCR alone without HI, SD, and PD outcomes were inferior to CR, PR, mCR+HI, and HI. In conclusion, CR by IWG 2006 response criteria can be used as a surrogate endpoint for OS in higher‐risk MDS pts. Any response associated with restoration of effective hematopoiesis is associated with better outcome.

## Graphical abstract

In higher risk MDS patients responses associated with restoration of effective hematopoiesis namely complete remission (CR) are associated with better outcomes.Marrow CR without hematological improvement outside context of allogeneic hematopoietic stem cell transplant should not be included in response criteria assessing new therapeutic agents in MDS.

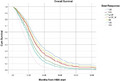

## 1 INTRODUCTION

The primary goal for treatment of higher‐risk myelodysplastic syndromes (MDS) patients is to improve overall survival (OS) and delay evolution to acute myeloid leukemia (AML).^1^ The criteria to assess response after MDS therapy were originally proposed by an International Working Group (IWG) of experts based on available data and consensus opinion in 2000 and were subsequently modified in 2006 (Table 1).^2^, ^3^

**TABLE 1:** IWG 2006 Response criteria

Category	Response Criteria (must last at least 4 weeks)
Complete Remission	Bone marrow: ≤5% myeloblasts with normal maturation of all cell lines Persistent dysplasia will be noted Hgb: ≥11 g/dL, platelets: ≥100 × 10^9^/L, neutrophils: ≥1.0 × 10^9^/L, blasts: 0%
Partial Remission	All CR criteria if abnormal before treatment except: Bone marrow blasts decreased by ≥50% over pretreatment but still >5% Cellularity and morphology not relevant
Marrow CR	Bone marrow: ≤5% myeloblasts and decrease by ≥50% over pretreatment Peripheral blood: if HI responses, they will be noted in addition to marrow CR
Stable Disease	Failure to achieve at least PR, but no evidence of progression for >8 wks
Disease progression	For patients with: Less than 5% blasts: 50% increase in blasts to 5% blasts 5%–10% blasts: 50% increase to 10% blasts 10%–20% blasts: 50% increase to 20% blasts 20%–30% blasts: 50% increase to 30% blasts Any of the following: At least 50% decrement from maximum remission/response in granulocytes or platelets Reduction in Hgb by 2 g/dL Transfusion dependence

The IWG 2006 response criteria are widely used in clinical trials to evaluate the efficacy of MDS treatments in a defined, systematic way. However, the IWG 2006 response criteria have not been accepted by health regulatory agencies as surrogate endpoints that clearly translate to clinically meaningful benefits, such as improved OS and delayed AML transformation. Moreover, in clinical practice, the use of IWG 2006 response criteria to determine treatment efficacy and patient benefit is not widespread. In an ad hoc landmark analysis of the AZA‐001 study in which higher‐risk MDS patients treated with azacitidine were compared to those treated with conventional care regimens, patients who achieved 2006 IWG‐defined responses of hematological improvement (HI), complete response (CR), or partial response (PR) demonstrated improved OS.^4^, ^5^ Increasingly, the response criterion *marrow CR* (mCR) has also been included in clinical trial measures of overall response rates. Whether this is associated with clinically meaningful endpoints has not been determined.

The utility of IWG 2006 response criteria outside of the context of clinical trials and their association with outcomes is unclear. Here, we assess the validity of the IWG 2006 response criteria, including mCR and SD, in a large cohort of higher‐risk MDS patients treated with multiple drugs at centers from the MDS Clinical Research Consortium (MDSCRC) and validated in a large separate cohort at Moffitt Cancer Center (MCC).

## 2 METHODS

Adult patients (>18 years) with higher‐risk MDS (ie, Intermediate‐2 [Int‐2] or High Risk by International Prognostic Scoring System^6^ [IPSS]) with diagnoses confirmed per World Health Organization criteria ^7^and who had received treatment and for whom details of response and outcome were available were included from MDSCRC centers. Follow‐up bone marrow assessments were obtained within 4–6 months of therapy initiation to assess response. Karyotyping was based on 20 metaphases with at least two cells expressing an abnormality required to define a clone. Patients were also reclassified per revised IPSS (IPSS‐R).^8^ The best response to treatment was categorized per the published IWG 2006 response criteria (Table 1) as CR, PR, mCR, HI, stable disease (SD), or progressive disease (PD).^3^ The responses were assessed by treating physicians and captured by own institutions database were pooled into the MDSCRC database. A separate cohort of high and very high‐risk IPSS‐R MDS patients treated at MCC was used for validation of results.

The primary endpoint was median OS and secondary endpoint was AML transformation, both measured from time start of therapy. For the MDSCRC cohort survival data were not censored for allogeneic transplant, the MCC patients selected cohort did not undergo transplant. The majority of front‐line treatments were the hypomethylating agents azacitidine or decitabine, alone or in combination with other drugs. Descriptive statistics were used for baseline characteristics. The Kaplan–Meier method was used to estimate OS and a log‐rank analysis was used to compare response categories. Cox regression analysis was used for multivariable analyses.. A two‐sided alpha <.05 defined significance.

## 3 RESULTS

We identified 646 treated IPSS higher‐risk MDS patients from the MDSCRC. Table 2 summarizes baseline characteristics. The median age was 68 years and the majority were Caucasian. Refractory anemia with excess blasts II (RAEB‐II) was the most common WHO subtype and one‐third were classified as therapy‐related MDS (t‐MDS). By IPSS stratification, two‐thirds of patients were intermediate‐2 risk, whereas half of the patients were very high risk by IPSS‐R. The first‐line treatment included a hypomethylating agent in 470 pts (74%): either azacitidine or decitabine monotherapy, or one of these two agents in combination with another drug. One‐third of the patients (29%) ultimately underwent allogeneic hematopoietic stem cell transplant.

**TABLE 2:** Baseline characteristics

Variable		MDS Clinical Consortium Original Cohort n = 646	MCC Cohort Validation Cohort n = 539
Age	Median	68	72
Gender	Male	399/645 (62%)	365/539 (68%)
Race	White	566/633 (89%)	489/539 (91%)
t‐MDS	Yes	161/545 (30%)	136/539 (25%)
WHO	RA	5/527 (1%)	0
	RARS	7/527 (1%)	0
	RCMD	69/527 (13%)	0
	RAEB‐I	153/527 (29%)	167 (31%)
	RAEB‐II	284/527 (54%)	300 (56%)
	MDS‐U	3/527 (1%)	1
	MDS/MPN	5/527 (1%)	0
	CMML	1/527 (1%)	0
	AML 20–30%	0	71 (13%)
IPSS	Intermediate‐II	468/646 (72%)	293 (54%)
	High	178/646 (28%)	246 (46%)
R‐IPSS	Very low	0	0
	Low	6/621 (1%)	0
	Intermediate	74/621 (12%)	0
	High	211/621 (34%)	178 (33%)
	Very High	330/621 (53%)	361 (46%)
IPSS karyotype	Good	135/642 (21%)	113 (21%)
	Intermediate	118/642 (18%)	104 (19%)
	Poor	389 /642 (61%)	320 (59%)
			Missing 2
R‐IPSS karyotype	Very good	7/642 (1%)	2
	Good	137/642 (21%)	116 (22%)
	Intermediate	134/642 (21%)	101 (19%)
	Poor	118/642 (18%)	85 (16%)
	Very poor	246/642 (38%)	231 (43%)
			Missing 4 (1%)
Allogeneic transplant	Yes	158/554 (29%)	Non transplant cohort
First‐line therapy	HMA	470/634 (74%)	539 (100%)
	Chemotherapy	57/634 (9%)	0
	IMiD	43/634 (7%)	0
	Clinical trial	25/634 (4%)	
	Other	38/634 (6%)	0
Lab (mean)	Hgb	9.2 g/dl	8.9 g/dl
	Platelets	92 × 109/L	86 × 109/L
	ANC	1.7 × 109/L	1.6 × 109/L
	Bone marrow blasts	10%	13%

The median duration of follow‐up from diagnosis was 16.2 months (mo); 95% CI: (15.2, 17.7). The median survival time from diagnosis was 18.0 mo; 95% CI: (16.7, 19.5). The median OS based on IPSS and IPSS‐R risk are summarized in Table 3 and was significantly worse for higher‐risk IPSS (*P* = 0.007) and IPSS‐R (*P* < 0.001) subgroups.

**TABLE 3:** Overall survival based on IPSS and IPSS‐R.

Risk Category	Median OS from Dx (mo)	95% CI	*P*‐Value
IPSS			0.007
Int‐2	18.7	17.3, 21.8	
High	15.5	13.9, 18.7	
IPSS‐R			<0.001
Low	72.5	27.0, NR	
Intermediate	39.1	23.8, 52.8	
High	23.6	18.9, 27.5	
Very High	14.3	13.5, 15.8	

**FIGURE 1:**
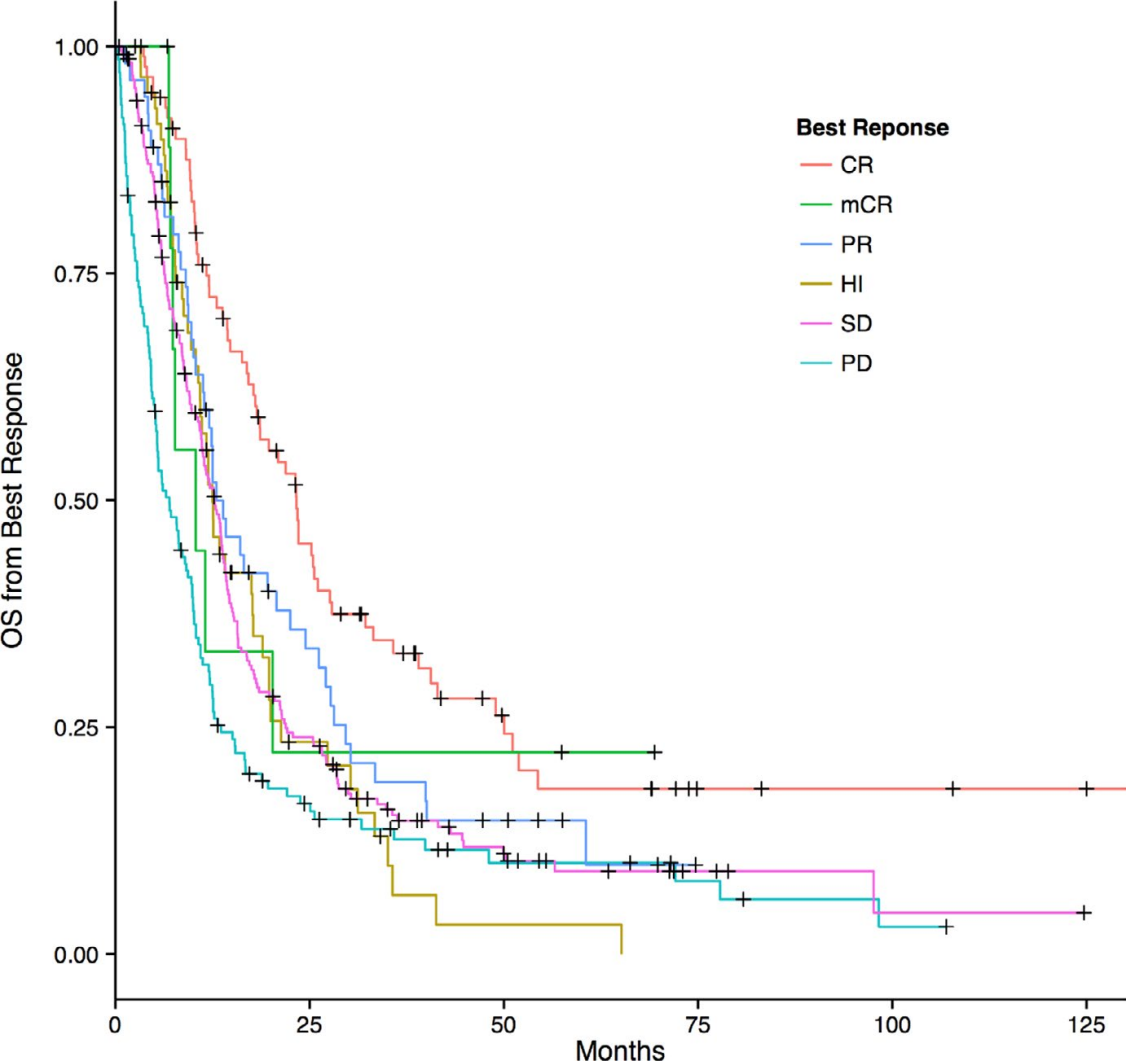
Median OS based on best response to first‐line therapy (MDSCC cohort)

The best response rates by IWG 2006 criteria to first‐line therapy were evaluable in 597 patients and included CR in 93 patients (16%), mCR in 10 (2%), PR in 57 (10%), HI in 60 (10%), SD in 239 (33%), and PD in 144 (24%). The median OS was 23.3 mo for those who achieved CR, 10.3 mo for mCR, 13 mo for PR, 12.5 mo for HI, 12.7 mo for SD, and 6.9 mo for PD. CR was associated with a better OS compared to all other response groups (*P* < 0.001) (Figure 1).

Among 580 patients evaluable for AML transformation, 310 (53%) transformed to AML. There was no difference in rate of AML transformation among response groups except in PD patients, who (as expected) transformed at a higher rate compared to others. The AML transformation rate was 52%, 46%, 43%, and 76% for CR, mCR/PR/HI, SD, and PD, respectively (*P* < 0.001). CR was associated with better leukemia‐free survival (LFS) compared to the mCR/PR/HI/SD combined group and compared to PD. Patients with mCR/PR/HI/SD had better LFS compared to those with PD. The LFS was 13.5, 7.8, 8.5, 10.3, 7.9, and 1.8 mo for CR, mCR, PR, HI, SD, and PD, respectively, *P* < 0.001.

Among 470 patients treated with HMA as first‐line therapy, response was evaluable in 448 pts. The overall Response Rate (ORR), defined as HI or better (CR/PR/mCR/HI) was 39%, similar to the ORR reported in the AZA‐001 and United States Intergroup (azacitidine monotherapy vs. azacitidine combined with lenalidomide or with vorinostat studies).^5^, ^9^ (Table 4) The median OS from time of starting therapy was 21 mo for CR, 8 mo for mCR, 14 mo for PR, 12 mo for HI, 13 mo for SD, and 8 mo for PD (*P* < 0.001). A CR was associated with better outcome compared to all other response groups. Patients with PR, HI, and SD had better outcome compared to PD. The median LFS was 16.3, 7.7, 8.6, 9.5, 7.9, and 2.3 mo for CR, mCR, PR, Hi, SD, and PD, respectively (*P* < 0.001).

**TABLE 4:** Response rate among higher‐risk MDS patients treated with HMA

IWG 2006 Response	n (%)
CR	68 (15)
mCR	8 (2)
PR	47 (10)
HI	54 (12)
SD	178 (40)
PD	93 (21)

In multivariable analyses, the best response by IWG 2006 criteria remained predictive of OS after adjusting for IPSS‐R risk group, HR 0.37 (95% CI 0.27– 0.51) for CR, and 0.64 (95% CI 0.51–0.80) for mCR/PR/HI/SD compared to PD, (*p* < 0.001). The best response by IWG 2006 criteria remained predictive of LFS after adjusting for IPSS‐R risk group, HR 0.20 (95% CI 0.14– 0.27) for CR, and 0.27 (95% CI 0.21–0.34) for mCR/PR/HI/SD compared to PD, (*p* < 0.001).

**FIGURE 2:**
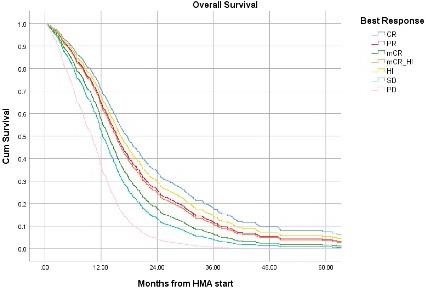
Overall Survival based on best response (MCC validation cohort)

We validated those findings in a separate cohort of high and very high IPSS‐R MDS patients treated at MCC who received HMA as first‐line therapy (Table 1). Among 539 patients who did not undergo allogeneic hematopoietic stem cell transplant, the best response to first‐line HMA was: CR in 90 (17%), PR in 6 (1%), mCR in 38 (7%), mCR with HI in 43 (8%), HI in 59 (11%), SD in 192 (36%), and PD in 111 (21%) patients, respectively. In multivariable Cox regression analysis after adjusting for IPSS‐R, mCR, SD, and PD were associated with significantly worse OS compared to CR. PR, mCR with HI, and HI. (Table 5 and Figure 2). The rate of AML transformation was 66%, 67%, 50%, 54%, 44%, 48%, and 88% for CR, PR, mCR, mCR+HI, HI, SD, an PD, respectively (*P* < 0.005). Only PD was associated with worse LFS after adjusting for IPSS‐R (Table 5).

**TABLE 5:** Hazard ratio for overall survival and leukemia‐free survival based on best response adjusted for IPSS‐R compared to CR among Moffitt Cancer Center Cohort (n = 539)

Response	n = 539	Overall Survival			Leukemia‐free Survival		
	n (%)	Hazard Ratio	95% CI	*P*‐value	Hazard Ratio	95% CI	*P*‐value
CR	90 (17%)						
PR	6 (1%)	1.25	0.5–3.1	0.6	1.4	0.5–3.9	0.4
mCR	38 (7%)	1.6	1.1–2.4	0.02	0.8	.5–1.4	0.5
mCR+HI	43 (8%)	1.3	0.9–1.9	0.18	0.85	.5–1.4	0.5
HI	59 (11%)	1.1	0.8–1.6	0.5	0.65	0.4–1.0	0.07
SD	192 (36%)	1.9	1.4–2.4	<0.005	.81	0.6–1.1	0.2
PD	111 (21%)	2.9	2.2–3.9	<0.005	1.8	1.3–2.6	<0.005

## 4 DISCUSSION

In this large retrospective study, we demonstrate that the best response by IWG 2006 criteria to first‐line therapy in higher‐risk MDS is associated with OS. This is particularly true for patients who achieved CR, which can be used as a surrogate for clinically meaningful outcomes. While patients who achieved SD or better response, as a combined group, had improved outcome compared to PD.

Within the AZA‐001 pivotal study, patients with stable disease had a survival advantage compared to those with progressive disease, but survival among those with stable disease did not differ between azacitidine or CCR. ^4^ We previously reported that among patients who had stable disease at 4–6 months after treatment initiation, 20% achieved a response later on, while those who achieved CR had superior OS compared to patients who remained with SD (28.1 vs. 14.4 months, respectively, *P* = 0.04).^10^

Patients with a mCR response achieved little clinical benefit to their treatment, as OS was worse than those with SD. The validation cohort from MCC corroborated those findings in a larger number of patients with a greater representation of mCR to specifically address this response category. An OS benefit was restricted to those patients with mCR who achieved HI only. Responses associated with restoration of effective hematopoiesis (CR, PR, mCR+HI, and HI) were associated with better outcome while mCR, SD, and PD were not. While this study is limited by its retrospective nature, it seems unlikely that using mCR as a response criterion in prospective trials will lead to demonstrable meaningful benefits to patients. Myeloblast reduction without restoration of some degree of hematopoiesis has not been demonstrated to improve outcomes. The ONTIME randomized clinical trial compared rigosertib to best supportive care. There were no CR or PR in both groups, the mCR was 20% in the rigosertib arm and 14% in the best supportive care. There was no overall survival benefit observed with rigosertib.^11^ The potential benefit of transient myeloblast reduction prior to allogeneic stem transplant could not be examined in this study.

There are challenges assessing mCR, HI, and SD outside of the context of clinical trials. For example, the ongoing assessment of transfusion reductions, and peripheral blood count improvement is limited by available data obtained during routine practice. The timing of bone marrow assessment after starting therapy is not standardized outside of trials. A landmark analysis at certain time points was not feasible in this retrospective study, as the clinicians and the database captured best response rather than responses at selected time points. Another limitation of our study is lack of central review or audit of response assessment. Still, the ORR reported herein aligned with two of the largest studies conducted in higher‐risk MDS, as did outcomes, indicating the validity of the endpoints assessed.

In conclusion, CR by IWG 2006 response criteria can be used as a surrogate endpoint for OS in higher‐risk MDS patients in randomized Phase II studies determining comparison arms of Phase III trials, and for regulatory purposes.

## CONFLICT OF INTEREST

Komrokji: *JAZZ*: Speakers Bureau and consultancy; *Novartis*: Speakers Bureau and consultancy; *Agios*: Speaker bureau and Consultancy; *Incyte*: Consultancy; *DSI*: Consultancy; *celgene*: Consultancy; *pfizer*: Consultancy, AbbVie Speakers Bureau and consultancy. Al Ali: no conflict of interest. Sallman: *Celgene*: Research Funding, Speakers Bureau; *Celyad*: Membership on an entity's Board of Directors or advisory committees; *Incyte*: Speakers Bureau; *Jazz*: Research Funding; *Novartis*: Speakers Bureau; *AbbVie*: Speakers Bureau. Padron: *Incyte*: Research Funding; *Kura Oncology*: Research Funding; *Celgene*: Research Funding. DeZern: *Astex Pharmaceuticals, Inc*.: Consultancy; *Celgene*: Consultancy. Barnard: no conflict of interest. Roboz:: Consultancy, Membership on an entity's Board of Directors or advisory committees; *Trovagene, Takeda, Sandoz, Roche/Genentech, Pfizer, Otsuka, Orsenix MEI Novartis, AbbVie, Actinium, Agios, Amphivena, Argenx, Astex, Astellas, Bayer, Celgene, Celltrion, Daiichi Sankyo, Eisai, Janssen, Jazz*. Garcia‐Manero: *Amphivena*: Consultancy, Research Funding; *Helsinn*: Research Funding; *Novartis*: Research Funding; *AbbVie*: Research Funding; *Celgene*: Consultancy, Research Funding; *Astex*: Consultancy, Research Funding; *Onconova*: Research Funding; *H3 Biomedicine*: Research Funding; *Merck*: Research Funding. List: *Celgene*: Membership on an entity's Board of Directors or advisory committees, Research Funding. Steensma: *Stemline*: Consultancy; *Pfizer*: Consultancy; *Aprea*: Research Funding; *H3 Biosciences*: Other: Research funding to institution, not investigator; *Astex*: Consultancy; *Arrowhead*: Equity Ownership; *Onconova*: Consultancy; *Summer Road*: Consultancy. Sekeres: no conflict of interest.

## AUTHORS CONTRIBUTION

RSK, DS, MS wrote manuscript, analyzed data, contributed patients; JB statistical analysis, NA data collection; EP, DS, AD, GR, GGM, AL contributed patients, reviewed manuscript. All authors approved final manuscript.

## ETHICAL APPROVAL

The study was approved by IRB. This was retrospective review.

## Data Availability

Data for this manuscript was extracted from MDS clinical research consortium and Moffitt cancer center MDS databases.
